# Comparison of computer-key-hold-time and alternating-finger-tapping tests for early-stage Parkinson’s disease

**DOI:** 10.1371/journal.pone.0219114

**Published:** 2019-06-27

**Authors:** Boon Leong Lan, Jacob Hsiao Wen Yeo

**Affiliations:** Electrical and Computer Systems Engineering & Advanced Engineering Platform, School of Engineering, Monash University, Bandar Sunway, Malaysia; Universitair Medisch Centrum Groningen, NETHERLANDS

## Abstract

Giancardo et al. recently introduced the neuroQWERTY index (nQi), which is a novel motor index derived from computer-key-hold-time data using an ensemble regression algorithm, to detect early-stage Parkinson’s disease. Here, we derive a much simpler motor index from their hold-time data, which is the standard deviation (SD) of the hold-time fluctuations, where fluctuation is defined as the difference between successive natural-log of hold time. Our results show the performance of the SD and nQi tests in discriminating early-stage subjects from controls do not differ, although the SD index is much simpler. There is also no difference in performance between the SD and alternating-finger-tapping tests.

## Introduction

Parkinson’s disease (PD) is a neurodegenerative disorder afflicting millions worldwide. Many methods have been proposed to assess PD objectively using various technologies. Generally, these methods are based on either motor or non-motor features. Motor features include axial features (such as balance and gait), bradykinesia, tremors, rigidity and speech; while examples of non-motor features are cognition, sleep and smell. A systematic review [1] of these methods published from 2005 to 2015 shows that axial features were studied most frequently, followed by bradykinesia (for example, through finger-tapping); while the most common technology used was inertial measurement units. To analyze gait, a variety of methods have been proposed, including detrended fluctuation analysis [2, 3], power spectra analysis [4], entropy analysis [5, 6] and machine-learning [7, 8].

In recent years, studies on PD assessment mostly continue to focus on the conventional features such as balance [9] and speech [10–12] for motor aspects, and cognition [13, 14] for non-motor aspects. However, there have also been studies on other non-motor aspects such as body fat distribution [15] and imaging biomarkers [16, 17].

For motor aspect, a novel method based on computer keystroke dynamics was introduced recently by Giancardo et al. [18] to detect early-stage PD. In particular, they derived a neuroQWERTY index (nQi) from the hold times (hold time is the time between pressing and releasing a key on a computer keyboard) using an ensemble regression algorithm, where the inputs are 7-element feature vectors, which include the skewness and histogram values of the hold times. More recently, Adams [19] derived a large number of keystroke features– 9 features from hold time and also 18 features from latency (which is the time interval between pressing one key and pressing the next key)–from a separate data set to detect early-stage PD using an ensemble of machine learning classification models. A test that could detect early-stage PD based on keystroke dynamics would be very easy to use since it does not need other specialized equipment and it could be self-administered at home by just typing on a computer keyboard.

Giancardo et al. [18] reported that their nQi test can discriminate early-stage PD from controls (the area under the ROC curve is 0.81), and the performance of the test is significantly better compared to the commonly-used alternating-finger-tapping (AFT) test. However, the nQi is a rather complex index, both in terms of how the 7-element feature vectors are defined and how the index is derived from the feature vectors. Here, we derive a new and much simpler motor index from Giancardo et al.’s hold-time data, which is simply the standard deviation (SD) of the hold-time fluctuations, and compare the performance of our SD test with the nQi and AFT tests to determine if the test performs better in discriminating early-stage PD from controls.

## Methods

The hold-time (HT) data of Giancardo et al. [18] is available from Physionet [20] as the neuroQWERTY MIT-CSXPD database. To derive the SD index for each subject from the HT data, we first take the natural-log of the hold times. Next, the difference between successive ln(HT)’s is calculated:

$$ln{\left(HT\right)}_{n+1}-ln{\left(HT\right)}_{n}=ln\frac{{\left(HT\right)}_{n+1}}{{\left(HT\right)}_{n}}.$$

We define these differences, which are dimensionless, as hold-time fluctuations. The SD index (given in S1 and S2 Tables as supporting information) is simply the standard deviation of the hold-time fluctuations. Following Giancardo et al. [18], HT for long metakeys (Shift, Ctrl and Alt), and backspace are not included for analysis; only HT for alphanumeric, symbol and space bar keys are included. Similar fluctuations were previously defined for heart-beat intervals [21] (where it was found that the spread of the RR-interval fluctuations can discriminate AF (atrial fibrillation) and congestive-heart-failure subjects from controls), animal [22] and marine microbial [23] population, and laser intensity [24].

Giancardo et al.’s nQi, AFT and single-key-tapping (SKT) data, which we also use here, were obtained from the same database in Physionet. We have checked that these data are the same as those published by them in [18] as supporting information, except the data for subject with ID 99 (a control), which is available in the database, is missing in their supporting information.

In Giancardo et al.’s [18] study, the subject alternatively pressed two buttons with the index finger for the AFT test. The test was repeated with the other index finger and the AFT score is the average number of presses. For the SKT test, the subject first repeatedly pressed a single button with the dominant hand. The test was repeated with the other hand and the SKT score is the average number of presses. 4 controls and 5 early-stage PD subjects do not have AFT data, but they have SKT data (see the supporting information in [18]).

For the typing test in Giancardo et al.’s study [18], the subject transcribed a folk tale on a standard word processor running on a standard computer. The HT data was collected by a software running in the background. This data is then divided into subsets using non-overlapping 90-seconds windows. From each subset of data, a 7-element feature vector is calculated. The first three features are, respectively, the percentage of outliers, the skewness of the data and a measure of finger coordination for two consecutive keystrokes. The last four features are, respectively, the values of the first four bins of an equally-spaced normalized histogram of the data. An ensemble regression algorithm (see [18] for the lengthy technical details] converts each feature vector to a numerical score. The nQi for each subject is the average of these numerical scores. Giancardo et al.’s multi-feature nQi is thus more complex than our single-feature SD index, and also fundamentally different because it does not incorporate changes in successive hold times. Moreover, converting the 7-element feature vectors to nQi requires a complex algorithm, whereas our SD index is easily obtained from the hold-time fluctuations.

All our statistical analyses were performed using the MedCalc statistical software [25]. In the ROC curve analysis, the 95% CI for the AUC is an exact Binomial confidence interval. Pairwise comparison of the ROC curves used the same method of DeLong et al. [26] as Giancardo et al. [18].

## Results

There are 43 control and 42 early-stage PD subjects in Giancardo et al.’s study [18]. All of the controls and early-stage PD subjects have nQi, AFT and SKT data, except 4 controls and 5 early-stage PD subjects who do not have AFT data. For a proper comparison of the AFT test with other tests, we therefore use the smaller set of 39 control and 37 early-stage PD subjects, who have all three data: nQi, AFT and SKT.

**Fig 1:**
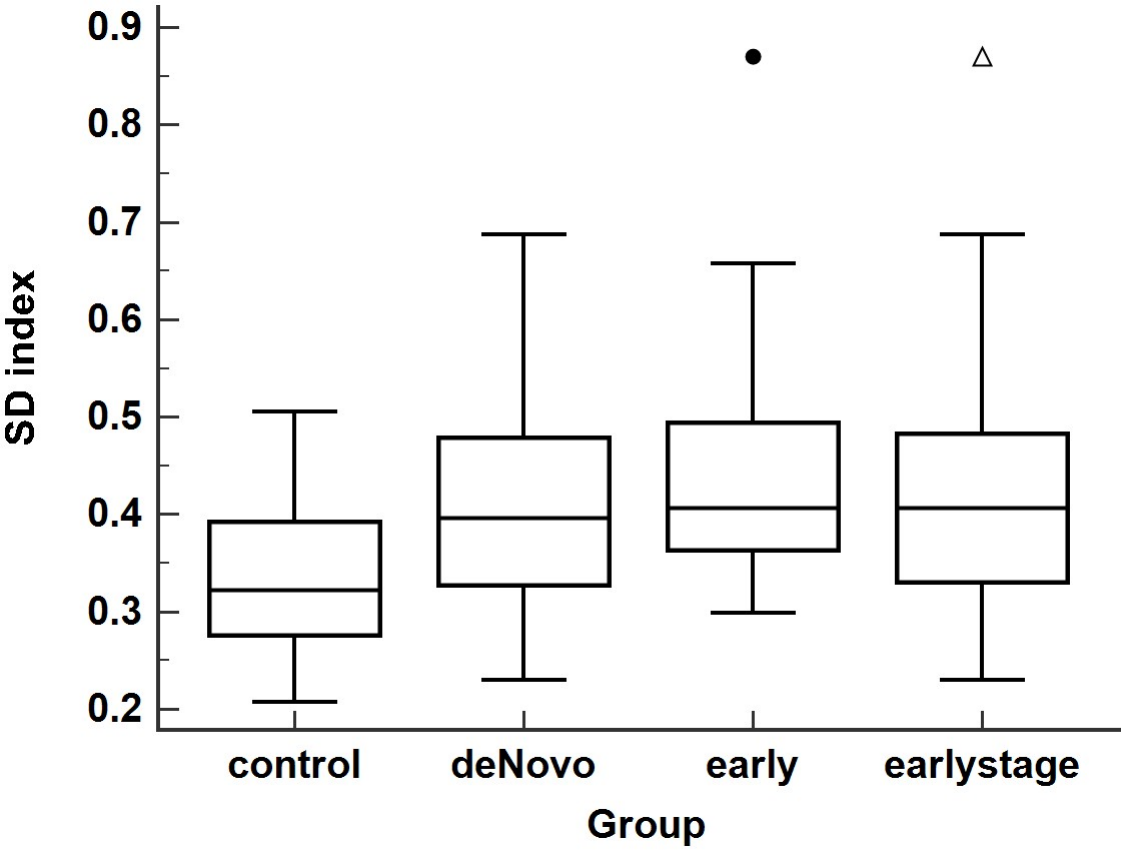
Box plots. Plots for the standard deviation (SD) of the hold-time fluctuations for the control group (n = 39), deNovo PD subgroup (n = 24), early PD subgroup (n = 13), and the early-stage PD group (n = 37).

The SD’s of the hold-time fluctuations (see Methods for definition) for the early-stage PD (37 subjects) and control (39 subjects) groups are significantly different (Mann-Whitney test; p = 0.0003). The SD’s for the deNovo PD and early PD subgroups are also significantly different from the controls (Mann-Whitney test; p = 0.0041 and p = 0.0023, respectively), but the SD’s for the two PD subgroups are not significantly different (Mann-Whitney test; p = 0.5041). Thus, we will not analyze the two subgroups separately, only the combined group referred to as early-stage will be analyzed. In the deNovo PD subgroup, the average time since diagnosis is 1.6 years, and the subjects have never taken PD medication [18]. In the early PD subgroup, the average time since diagnosis is 3.9 years, and the subjects are on PD medication but not 18 hours prior to the typing test [18]. Box plots of the SD’s for every group and subgroup are shown in Fig 1.

As a check, for the nQi and SKT tests, the area under the ROC curve (AUC) and corresponding 95% confidence-interval (CI) we obtained for the full set of 43 control and 42 early-stage PD subjects agree with those of Giancardo et al. [18]. The AUC we obtained is 0.807 (CI 0.707 to 0.884) for nQi, and 0.609 (CI 0.497 to 0.713) for SKT. The AUC they [18] obtained is 0.81 (CI 0.72 to 0.88) for nQi, and 0.61 (CI 0.51 to 0.71) for SKT.

For the AFT test (39 control, 37 early-stage PD), Giancardo et al. [18] originally reported 0.75 (CI 0.64 to 0.83) for the AUC, but they [27] subsequently reported a corrected value of 0.83 (CI 0.75 to 0.91). The AUC and corresponding CI we obtained (see Table 1) agree with their corrected values. The AUC, CI and significance level we obtained for the nQi, SD and SKT tests, for the same subjects (39 control, 37 early-stage PD) as the AFT test, are also given in Table 1. The AUC is significantly different from 0.5 only for the nQi, SD and AFT tests, and thus these three tests are able to discriminate early-stage PD cases from normal cases.

**Table 1:** ROC curve analysis.

Test	AUC	95% CI	Significance level
nQi	0.789	0.681 to 0.874	<0.0001
SD	0.741	0.628 to 0.835	<0.0001
AFT	0.833	0.730 to 0.908	<0.0001
SKT	0.605	0.486 to 0.715	0.1171

Area under the ROC curve (AUC), 95% confidence interval (CI) for the AUC, and significance level for the nQi, SD, AFT and SKT tests. For every test, the sample size is 76 (control = 39, early-stage PD = 37).

Pairwise comparison of the ROC curves for the nQi, SD and AFT tests show that the AUC’s are not significantly different (see Table 2). Thus, there is no difference in the performance of the SD test compared with nQi test (although the SD is a much simpler index compared to the nQi) and also the AFT test. There is also no difference in the performance between the nQi and AFT tests, in agreement with Giancardo et al.’s [27] corrected result.

**Table 2:** Comparison of ROC curves.

Tests compared	Difference between AUC’s	Significance level
SD and nQi	0.0485	0.5014
SD and AFT	0.0918	0.2619
nQi and AFT	0.0433	0.5090

Pairwise comparison of areas under the ROC curves (AUC) for the nQi, SD and AFT tests. In all cases, the sample size is 76 (control = 39, early-stage PD = 37).

For the full set of 43 control and 42 early-stage PD subjects, the AUC is 0.752 and the CI is 0.647 to 0.840 for the SD test, which are close to the values for the smaller set of 39 control and 37 early-stage PD subjects (see Table 1). For the nQi test, the AUC and CI (given above) for the full set of subjects are also close to those for the smaller set of subjects (see Table 1). For the full set of subjects, the AUC is also significantly greater than 0.5 for both the SD and nQi tests (p<0.0001). However, the AUC’s for these two tests are still not significantly different—the difference between the areas is 0.0543 (p = 0.4073). In other words, there is also no difference in the performance of the SD and nQi tests for the full set of subjects.

## Conclusion and discussion

Our result shows there is no difference in the performance of the SD and nQi tests in discriminating early-stage PD from controls although the SD index is much simpler. Our result also shows the performance of the SD and AFT tests do not differ.

Milne and co-authors [28] have also utilized Giancardo et al.’s hold-time data in a recent study. Similar to our work here, they proposed a single-feature approach to detect early-stage PD. In one approach, their Stdev index is the standard deviation of the hold times. However, their Stdev index is different from our SD index, which is the standard deviation of the differences between consecutive natural-log’s of hold time (see Methods). In another approach, their MACD index is the mean of the absolute differences between consecutive hold times. Clearly, their MACD index and our SD index are also different. Milne and co-authors reported that the performance of their Stdev test (AUC = 0.82) is ‘comparable’ to Giancardo et al.’s nQi test (AUC = 0.81). They also reported that the MACD test (AUC = 0.85) ‘significantly’ outperformed the Stdev and nQi tests. However, they did not report the p values to support the reported statistical significance of the pairwise difference between the AUC’s for the nQi, Stdev and MACD tests. Furthermore, Milne and co-workers did not compare the performance of their Stdev and MACD tests with the AFT test.

Giancardo et al.’s dataset is small (43 control and 42 early-stage PD). To mitigate the risk of overfitting, Giancardo et al. employed a cross-training strategy using two subsets, deNovo and early-PD. The ensemble regression model trained on one subset is used to generate the nQi’s for the other subset, and vice versa. Milne and co-workers [28] also used the same cross-training strategy in their logistic regression model. In our case, overfitting is not an issue since there is no model fitting—the SD index for each subject is the standard deviation of the hold-time fluctuations for the subject. The performance of all these tests in discriminating early-stage PD from controls should of course be validated with a larger dataset. However, this cannot be done at present since the only other existing dataset, as far as we know, that contains hold-time data is Adams’ [19], but it is even smaller (33 control and 20 early-stage PD) and there is no alternating-finger-tapping data.

The diagnosis of PD is currently still based on subjective clinical observation, which could result in misdiagnosis. One study [29] shows that the sensitivity and specificity is 93.5% and 64.5%, respectively, for clinical diagnosis by specialists, which has a high rate of false positive. In comparison, the nQi, SD, Stdev and MACD hold-time tests have higher specificity (80%, 80%, 86%, 81% respectively) but lower sensitivity (70%, 57%, 64%, 81% respectively) than the diagnosis by specialists. Whether it is possible to achieve an AUC close to 1, with very high sensitivity and also specificity, using a single-feature test derived from hold-time data to discriminate early-stage PD from controls remains to be investigated. However, we are not suggesting that such a test would replace a trained clinician diagnosis but rather as an adjunct to help support a clinical diagnosis.

## Supporting information

S1 Table SD index. Standard deviation (SD) of the hold-time fluctuations for the early PD subgroup (diagnosis = 1) and control (diagnosis = 0).
(DOCX)

S2 Table SD index. Standard deviation (SD) of the hold-time fluctuations for the deNovo PD subgroup (diagnosis = 1) and control (diagnosis = 0).
(DOCX)
