# Supplementary material for: Comparison of computer-key-hold-time and alternating-finger-tapping tests for early-stage Parkinson’s disease
Source: PLoS One. 2019 Jun 27;14(6):e0219114. doi: 10.1371/journal.pone.0219114 (PMC6597101; doi:10.1371/journal.pone.0219114)
Supplement: S1 Table — Standard deviation (SD) of the hold-time fluctuations for the early PD subgroup (diagnosis = 1) and control (diagnosis = 0). (DOCX) [file pone.0219114.s001.docx]

| **ID** | **Diagnosis** | **SD index** |
| --- | --- | --- |
| 11 | 1 | 0.562101815 |
| 60 | 0 | 0.419674025 |
| 67 | 1 | 0.437240843 |
| 68 | 0 | 0.229537496 |
| 70 | 1 | 0.326283757 |
| 71 | 1 | 0.329574574 |
| 72 | 1 | 0.514865409 |
| 73 | 1 | 0.484808616 |
| 74 | 1 | 0.429564532 |
| 75 | 1 | 0.298934 |
| 76 | 0 | 0.366436167 |
| 77 | 0 | 0.283029008 |
| 78 | 1 | 0.374255831 |
| 79 | 0 | 0.439853962 |
| 80 | 1 | 0.475037028 |
| 81 | 0 | 0.417674566 |
| 82 | 1 | 0.405909592 |
| 83 | 0 | 0.286063311 |
| 84 | 1 | 0.522339536 |
| 85 | 0 | 0.505539427 |
| 86 | 1 | 0.317244086 |
| 87 | 0 | 0.357223159 |
| 88 | 1 | 0.402215361 |
| 89 | 0 | 0.313920266 |
| 92 | 1 | 0.869995236 |
| 93 | 1 | 0.431972866 |
| 94 | 0 | 0.431184605 |
| 95 | 0 | 0.439038709 |
| 97 | 1 | 0.406114809 |
| 98 | 1 | 0.657392586 |
| 99 | 0 | 0.274546033 |
