# Supplementary material for: Comparison of computer-key-hold-time and alternating-finger-tapping tests for early-stage Parkinson’s disease
Source: PLoS One. 2019 Jun 27;14(6):e0219114. doi: 10.1371/journal.pone.0219114 (PMC6597101; doi:10.1371/journal.pone.0219114)
Supplement: S2 Table — Standard deviation (SD) of the hold-time fluctuations for the deNovo PD subgroup (diagnosis = 1) and control (diagnosis = 0). (DOCX) [file pone.0219114.s002.docx]

| **ID** | **Diagnosis** | **SD index** |
| --- | --- | --- |
| 1000 | 1 | 0.38374599 |
| 1001 | 1 | 0.370233178 |
| 1002 | 0 | 0.348576446 |
| 1004 | 1 | 0.357151704 |
| 1005 | 1 | 0.229825117 |
| 1006 | 1 | 0.421316687 |
| 1008 | 1 | 0.589070489 |
| 1009 | 1 | 0.361564838 |
| 1010 | 0 | 0.295473643 |
| 1011 | 0 | 0.31530293 |
| 1012 | 0 | 0.262002607 |
| 1013 | 0 | 0.322765545 |
| 1014 | 1 | 0.568884593 |
| 1015 | 0 | 0.250375306 |
| 1016 | 0 | 0.433060807 |
| 1017 | 1 | 0.33109066 |
| 1019 | 1 | 0.474510235 |
| 1020 | 1 | 0.457381894 |
| 1021 | 0 | 0.255846263 |
| 1022 | 0 | 0.381552203 |
| 1023 | 1 | 0.481714169 |
| 1024 | 1 | 0.354039284 |
| 1025 | 1 | 0.687482844 |
| 1028 | 1 | 0.475578565 |
| 1029 | 0 | 0.34065333 |
| 1030 | 0 | 0.35237355 |
| 1031 | 0 | 0.386023804 |
| 1032 | 0 | 0.393471169 |
| 1033 | 0 | 0.405410727 |
| 1034 | 1 | 0.315189299 |
| 1035 | 0 | 0.393778004 |
| 1037 | 1 | 0.518825412 |
| 1039 | 0 | 0.258220239 |
| 1041 | 1 | 0.321355693 |
| 1042 | 0 | 0.311273569 |
| 1043 | 0 | 0.218339631 |
| 1045 | 0 | 0.336396523 |
| 1047 | 1 | 0.485112306 |
| 1049 | 0 | 0.278915209 |
| 1050 | 0 | 0.219633672 |
| 1051 | 0 | 0.2712559 |
| 1052 | 1 | 0.408912865 |
| 1053 | 0 | 0.229279633 |
| 1055 | 0 | 0.32171642 |
| 1056 | 0 | 0.389540925 |
| 1057 | 0 | 0.207529315 |
| 1059 | 1 | 0.323606408 |
| 1061 | 0 | 0.281803019 |
| 1062 | 0 | 0.301850367 |
| 1063 | 0 | 0.490346635 |
| 1064 | 1 | 0.458366652 |
| 1066 | 1 | 0.311101294 |
| 1068 | 1 | 0.266569365 |
| 1070 | 0 | 0.412295012 |
